# Effects of an embodied music intervention on executive function, emotion regulation and psychophysiological well-being in children: the role of movement-rhythm synchronization and self-regulation

**DOI:** 10.3389/fpsyg.2026.1988460

**Published:** 2026-10-20

**Authors:** Qian Shu

**Affiliations:** School of Music and Dance, Yibin University, Yibin, China

**Keywords:** children, embodied music, emotion regulation, executive function, psychological well-being, self-regulation, sensorimotor synchronization

## Abstract

Music participation engages auditory processing, temporal prediction, attention, and motor coordination, but the additional contribution of structured bodily engagement to children’s cognitive and emotional development remains unclear. This randomized controlled trial examined whether a 12-week embodied music intervention produced greater changes in executive function, emotion regulation, movement-rhythm synchronization, and psychophysiological well-being than non-embodied music participation or usual activity. A total of 180 children aged 8–11 years were randomly assigned to embodied music (*n* = 60), non-embodied music (*n* = 60), or usual-activity control (*n* = 60). The two music conditions were matched for duration, musical exposure, instructor contact, and session frequency; the embodied condition additionally incorporated whole-body rhythmic coordination, movement inhibition, rule switching, spatial movement, and cooperative synchronization. Assessments were conducted at baseline (T0), week 6 (T1), week 12/post-intervention (T2), and 3-month follow-up (T3). Primary outcomes were executive function and emotion regulation; secondary outcomes included movement-rhythm synchronization, psychological well-being, resting heart rate variability indexed by RMSSD, and sleep problems. Linear mixed-effects models showed significant Group x Time interactions for executive function, chi-square(6) = 78.60, *p* < 0.001; emotion regulation, chi-square(6) = 51.40, *p* < 0.001; and movement-rhythm synchronization, chi-square(6) = 129.30, *p* < 0.001. At T2, embodied music showed larger model-estimated improvements than non-embodied music in executive function [adjusted difference in change = 6.40, 95% CI (3.80, 9.00), *p* < 0.001], emotion regulation [4.30, 95% CI (2.00, 6.60), *p* < 0.001], and movement-rhythm synchronization [10.90, 95% CI (8.20, 13.60), *p* < 0.001]. Benefits were largely maintained at follow-up. Changes in psychological well-being and RMSSD were more moderate, and sleep effects were smaller. Exploratory indirect-effect analyses were statistically consistent with the hypothesized association among embodied music, synchronization, executive function, emotion regulation, and later well-being [standardized serial indirect effect = 0.022, bootstrap 95% CI (0.009, 0.043)]; because the proposed mediators were not fully separated in time, these analyses do not establish temporal or causal mediation. Overall, the randomized comparisons support benefits of embodied music for several proximal outcomes, while the proposed mechanisms require further confirmatory study.

## 1 Introduction

Executive function includes inhibition, working memory, and cognitive flexibility, which develop substantially during childhood and support learning, adjustment, and self-regulation (Miyake et al., 2000; Diamond, 2013). These control processes also contribute to emotion regulation by supporting response inhibition, attentional shifting, and maintenance of regulatory goals (Etkin et al., 2015). Because childhood self-regulation predicts later psychological and educational outcomes (Robson et al., 2020), interventions that engage cognitive, emotional, and behavioral regulation may have broad developmental relevance.

Music provides a potentially useful training context because active participation requires attention, temporal prediction, memory for rhythmic structure, response inhibition, and rapid behavioral adjustment. Experimental and longitudinal studies have reported associations between music training and children’s executive function (Moreno et al., 2011; Jaschke et al., 2018; Shen et al., 2019), although reviews emphasize substantial heterogeneity across training formats, outcome measures, and control conditions (Rodriguez-Gomez and Talero-Gutierrez, 2022; Sala and Gobet, 2020; Lu et al., 2025). This heterogeneity suggests that the cognitive demands of music training may depend partly on how strongly perception is coupled to action.

Embodied music cognition provides a framework for this distinction. Bodily movement can shape musical perception and representation, while synchronization with an external beat requires temporal prediction, motor planning, and error correction (Barsalou, 2008; Maes et al., 2014; Repp and Su, 2013). Developmental evidence also indicates that movement can influence rhythmic perception (Phillips-Silver and Trainor, 2005). Accordingly, embodied rhythmic activities repeatedly recruit inhibition, rule maintenance, switching, and coordinated action; rhythm-focused training has shown promise for inhibitory control (Frischen et al., 2019).

Embodied music may also relate to emotional and social regulation. Joint music making has been associated with cooperation and prosocial behavior (Kirschner and Tomasello, 2010), and sustained group music interaction has been linked to empathy (Rabinowitch et al., 2013). More distal outcomes may include psychological well-being and autonomic regulation. Resting HRV, particularly RMSSD, is commonly used as an index related to vagal regulation and flexible psychophysiological control (Task Force of the European Society of Cardiology and the North American Society of Pacing and Electrophysiology, 1996; Laborde et al., 2017; Thayer et al., 2012), although such outcomes are less directly trained than synchronization or executive control.

Three gaps motivated the present trial: many previous studies have lacked an active music control, few have combined executive, emotional, and physiological outcomes, and potential pathways linking embodied rhythmic participation with broader well-being remain under-tested. We therefore compared a 12-week embodied music intervention with a non-embodied active music condition matched for musical exposure, instructor contact, and duration, as well as with usual activity. The embodied condition additionally emphasized whole-body synchronization, response inhibition, movement-rule switching, and cooperative coordination.

We hypothesized that embodied music would produce greater gains in executive function (H1) and emotion regulation (H2), more modest changes in psychological well-being and autonomic regulation (H3), and the largest effect on directly trained movement-rhythm synchronization (H4). We further hypothesized an association between synchronization change and executive-function improvement (H5) and explored whether a serial indirect-effect pattern involving synchronization, executive function, and emotion regulation was statistically associated with later psychological well-being (H6).

## 2 Materials and methods

### 2.1 Research design

This three-arm, parallel-group randomized controlled trial used a 1:1:1 allocation ratio and four assessment occasions: baseline (T0), week 6 (T1), week 12/post-intervention (T2), and 3-month follow-up (T3). Children were randomized individually within each school to embodied music, non-embodied active music, or usual activity; intervention participants subsequently attended fixed school-by-condition cohorts. Outcome assessors were blinded to allocation. Reporting was guided by CONSORT 2025 recommendations (Hopewell et al., 2025). The trial was not prospectively registered; the dated protocol and analysis plan are available upon request. Primary outcomes and principal longitudinal comparisons are identified in the Methods, whereas the serial indirect-effect analysis was exploratory. School staff supported recruitment and scheduling, and parents or legal guardians completed consent procedures.

### 2.2 Participants

Children aged 8–11 years were recruited from six public primary schools in Tianjin, China. Screening and baseline assessment occurred from 12 January to 6 February 2026, the intervention from 23 February to 15 May 2026, and follow-up from 10 to 21 August 2026. Eligibility required primary-school enrollment, age 8–11 years, ability to follow task instructions, parental or legal-guardian consent, and child assent. Exclusion criteria were neurological or severe developmental conditions that precluded participation or assessment, concurrent music intervention of comparable intensity, or physical limitations preventing safe whole-body movement. Participating schools provided appropriate space, scheduling support, and access to qualified instructors who completed the study training and fidelity procedures.

Of 214 children screened, 180 were randomized equally to embodied music, non-embodied music, and usual activity (*n* = 60 per group). Outcome data were available for 176 participants at T1 (59/59/58 by group), 172 at T2 (58/57/57), and 166 at T3 (56/55/55), corresponding to 92.2% retention at final follow-up (Figure 1). All 180 randomized participants were retained in the intention-to-treat mixed-model analyses through use of their available repeated observations.

**Figure 1:**
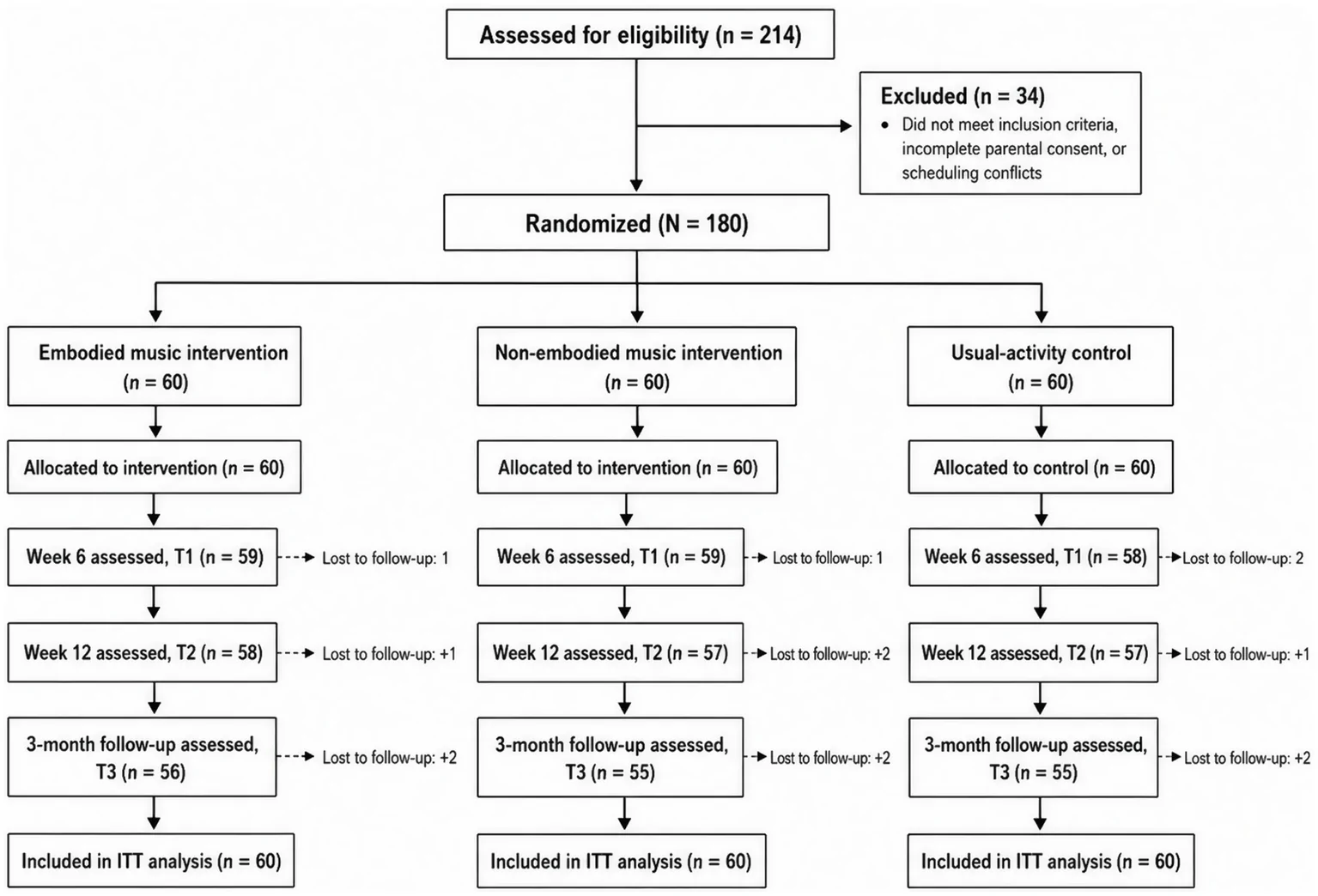
Participant flow diagram. Of 214 children screened, 180 underwent randomization to embodied music (*n* = 60), non-embodied music (*n* = 60), or usual-activity control (*n* = 60). Follow-up data were available for 176 participants at week 6, 172 at week 12 and 166 at the 3-month follow-up. All randomized participants were retained in intention-to-treat analyses.

An *a priori* G*Power 3.1.9.7 analysis for the repeated-measures Group x Time interaction assumed *f* = 0.15, alpha = 0.05, power = 0.90, three groups, four occasions, a repeated-measures correlation of 0.55, and epsilon = 0.75. The value *f* = 0.15 was used as a conservative small-to-moderate planning assumption rather than being estimated from the observed study data; it lies between Cohen’s conventional benchmarks for small (*f* = 0.10) and medium (*f* = 0.25) effects (Cohen, 1988). The resulting minimum analyzable sample was 153 participants. Allowing approximately 15% attrition yielded the target enrollment of *N* = 180 (60 per group). No interim efficacy analyses or stopping rules were specified. This calculation did not explicitly model school-by-condition clustering and should therefore be interpreted as an individual-level planning calculation rather than a cluster-adjusted power analysis.

### 2.3 Randomization and allocation

After baseline assessment, children were randomized within school and sex strata to one of the three groups using computer-generated permuted blocks of 3 and 6, so each school contributed participants to all study conditions. An independent statistician with no role in recruitment, intervention delivery, outcome assessment, or data collection generated the allocation sequence in R 4.3.2. A separate administrator prepared sequentially numbered, opaque, sealed envelopes that were opened only after eligibility and baseline assessment had been completed. Recruiters and baseline assessors did not have access to the allocation list, and intervention conditions were scheduled separately to reduce contamination. School-specific allocation counts are provided in Supplementary Table S1; baseline characteristics are summarized descriptively in Table 1.

**Table 1:** Baseline characteristics of the randomized sample.

Variable	Embodied music (*n* = 60)	Non-embodied music (*n* = 60)	Control (*n* = 60)
Age, years	9.52 +/− 1.02	9.48 +/− 1.05	9.55 +/− 1.00
Girls, *n* (%)	31 (51.7)	30 (50.0)	29 (48.3)
Prior music training, *n* (%)	17 (28.3)	18 (30.0)	16 (26.7)
Physical activity, days/week	3.41 +/− 1.19	3.32 +/− 1.14	3.39 +/− 1.17
Executive function composite	50.2 +/− 8.0	50.0 +/− 7.9	49.8 +/− 8.1
Emotion regulation composite	48.5 +/− 7.2	48.7 +/− 7.1	48.4 +/− 7.3
Movement-rhythm synchronization	50.1 +/− 8.5	50.0 +/− 8.4	49.9 +/− 8.6
RMSSD, ms	38.5 +/− 11.0	38.7 +/− 11.2	38.4 +/− 11.1

Values are mean +/− SD unless otherwise stated. Baseline characteristics are presented descriptively; no hypothesis tests are used to infer group equivalence.

### 2.4 Intervention duration and dose

Both active conditions lasted 12 weeks and comprised three 45-min sessions per week. Task difficulty was increased progressively across the intervention period.

Within each school, randomized children in the two active arms attended fixed, separately taught cohorts of approximately 8–12 children; controls received no additional sessions. Randomization occurred at the individual level, but intervention delivery created shared school-by-condition exposure, which was represented in the multilevel modeling strategy.

### 2.5 Embodied music intervention

The embodied program integrated rhythm, whole-body movement, inhibitory control, rule switching, and interpersonal synchronization. Each 45-min session comprised approximately 5 min of warm-up/body awareness, 8 min of beat synchronization, 10 min of rhythm-movement mapping, 8 min of inhibitory-control games, 8 min of rule switching, 4 min of paired/group synchronization, and 2 min of cool-down. Activities included clapping, stepping, body percussion, stopping or reversing actions in response to musical cues, and adapting movement to changes in rhythm, tempo, instrument, or rule. The program was designed as perception-action and executive-control training rather than dance instruction.

### 2.6 Non-embodied music active control

The non-embodied group received the same session frequency and duration and comparable musical materials. Activities included guided listening, singing, melody and pitch discrimination, rhythm identification, musical storytelling, and discussion. Whole-body locomotion, sustained sensorimotor synchronization, and movement-based rule switching were minimized by design. This active-control condition was intended to match instructor attention, musical exposure, novelty, social participation, and intervention duration while reducing the structured bodily component that distinguished the embodied condition.

Six qualified music instructors participated, one per school. Each held at least a bachelor’s qualification in music education or a related field and had at least 3 years of primary-school teaching experience. To reduce confounding by instructor, the same instructor delivered both active conditions within each school in separate sessions using condition-specific manuals. Instructors completed an 8-h centralized workshop and a 4-h supervised training session. Standardized session structures, matched intervention dose, shared core musical materials where appropriate, and regular supervision were used to support consistency across schools.

### 2.7 Usual-activity control

Children assigned to the usual-activity control condition continued their standard school activities and did not receive an additional music program during the intervention period. They completed outcome assessments at the same four time points as the two intervention groups.

### 2.8 Primary outcome: executive function

Executive function was assessed using inhibition, working-memory, and cognitive-flexibility tasks. Inhibition was measured with a child arrow Flanker task (Eriksen and Eriksen, 1974; 16 practice and 80 test trials). Responses shorter than 200 ms or longer than 2,500 ms were excluded, and inverse-efficiency scores (IES = mean correct reaction time/proportion correct) were calculated following Bruyer and Brysbaert (2011). The incongruent-minus-congruent IES was reverse-oriented so that higher values reflected better inhibition after reaction time and accuracy were inspected separately. Working memory was assessed using backward digit span (Wechsler, 2014; two trials per span, 2–8 digits, discontinued after two failures), scored as the number of correct sequences. Cognitive flexibility was assessed with a cued switching task adapted from Rogers and Monsell (1995; 20 practice and 80 test trials); switch-minus-repeat IES was reverse-oriented. These direction reversals and standardization procedures were study-specific analytic transformations rather than modifications to the original task instructions. Direction-aligned indices were converted to pooled-baseline T-scores (T = 50 + 10z), and their mean formed the executive-function composite.

### 2.9 Primary outcome: emotion regulation

Emotion regulation was assessed at each wave with the parent-reported Emotion Regulation Checklist (Shields and Cicchetti, 1997), with the same parent or guardian used across waves where feasible. Dysregulation-oriented items were reverse-coded before forming the reported composite so that higher scores indicated more adaptive regulation. Because the present report uses an overall composite rather than the original subscale structure, this score should be interpreted as a study-specific summary derived from the parent-reported item set.

### 2.10 Movement-rhythm synchronization

Movement-rhythm synchronization was assessed with a sensorimotor synchronization procedure informed by the literature reviewed by Repp and Su (2013), involving finger tapping and/or stepping in time with musical or metronomic beats. Mean absolute asynchrony and within-participant variability were direction-aligned and standardized before being combined so that higher scores indicated more accurate and stable synchronization.

### 2.11 Psychological well-being

Psychological well-being was assessed with the KIDSCREEN-10 health-related quality-of-life index (Ravens-Sieberer et al., 2010), reported as standardized T-scores, with higher values indicating better well-being.

### 2.12 Heart rate variability

Resting HRV was recorded during a standardized 5-min seated rest. RMSSD (ms) was used as the primary time-domain index because of its relevance to parasympathetic cardiac regulation (Laborde et al., 2017). Recordings underwent artifact inspection and preprocessing.

### 2.13 Sleep problems

Sleep problems were assessed by parent report using the Children’s Sleep Habits Questionnaire framework (Owens et al., 2000), with higher scores indicating greater sleep difficulty. Sleep was treated as a secondary, distal outcome.

Sample-specific reliability estimates were acceptable to good. Across waves, Emotion Regulation Checklist alpha/omega ranged 0.88–0.90/0.89–0.91; KIDSCREEN-10, 0.84–0.86/0.85–0.87; and sleep, 0.81–0.83/0.82–0.84. Executive-function composite omega ranged 0.82–0.86; split-half reliability was 0.83–0.85 for the Flanker task and 0.81–0.84 for rule switching; backward digit-span KR-20 was 0.79–0.82 (Supplementary Table S2).

### 2.14 Covariates

Baseline covariates were age, sex, prior structured music learning, and habitual physical activity because each could plausibly relate to cognitive or movement performance.

### 2.15 Intervention adherence and fidelity

Attendance averaged 91.8% in the embodied condition and 92.4% in the non-embodied condition (*p* = 0.64). Of 432 active-condition sessions, 86 (19.9%) underwent independent fidelity observation across conditions, schools, and intervention phases. Two trained assistants conducted the observations; inter-rater reliability for sessions rated by both observers was ICC = 0.92. Because embodied and non-embodied sessions were visibly distinguishable, fidelity observers were not blinded to treatment condition. They were independent of intervention delivery and outcome assessment. Mean fidelity across the two active conditions was 94.6% (SD = 3.8). Harms were monitored after sessions and at T1–T3: four embodied and one non-embodied participant reported transient soreness or fatigue that resolved without treatment; no serious adverse events, medically treated injuries, or harm-related withdrawals occurred.

### 2.16 Statistical analysis

Continuous and categorical variables were summarized using means/SDs and frequencies/percentages, respectively. Baseline characteristics were presented descriptively; inferential baseline significance tests were not used to establish group equivalence. Primary longitudinal outcomes were analyzed using multilevel linear mixed-effects models with repeated observations nested within participants and participants associated with school-by-condition cohorts. The model specification corresponded to Outcome ~ Group * Time + School + (1 | Participant) + (1 | Cohort), with Group × Time as the principal efficacy test. Follow-up analyses included all three pairwise group contrasts (Embodied vs. Non-embodied, Embodied vs. Control, and Non-embodied vs. Control) at each follow-up occasion, with Holm adjustment applied across the three comparisons within each outcome/time family. Postbaseline-versus-T0 contrasts were used to describe within-group change but were not interpreted alone as evidence of intervention efficacy.

Sensitivity models adjusted for age, sex, baseline physical activity, and prior music training; omitting the cohort random intercept did not change the substantive conclusions, although this sensitivity analysis does not by itself resolve the limited number of higher-level school-by-condition cohorts. Analyses followed the intention-to-treat principle. Missing outcomes were not singly imputed; likelihood-based mixed-effects models used all available observations under a missing-at-random assumption. T3 data were available for 56/60, 55/60, and 55/60 participants by group (Figure 1). Selected T2 between-group differences were summarized descriptively using Cohen’s d; these endpoint standardized effects do not account for the full repeated-measures and clustering structure and are secondary to the longitudinal model-based contrasts. Analyses used R 4.4.1, lme4 1.1–35.5, lmerTest 3.1–3, emmeans 1.10.4, and effectsize 0.8.9.

Exploratory indirect-effect analyses examined whether the embodied-versus-non-embodied contrast was statistically associated with later well-being through synchronization, executive function, and emotion regulation. Models were estimated in lavaan 0.6–19 using 5,000 bootstrap resamples for indirect-effect confidence intervals. Because synchronization, executive function, and emotion regulation were represented largely by overlapping T0-to-T2 changes and were not experimentally manipulated or fully separated in time, these analyses were treated as exploratory associations rather than evidence of serial temporal or causal mediation. A two-sided *p* < 0.05 threshold was used for the reported analyses; the multiplicity adjustment described above applied to the planned pairwise outcome contrasts, not to the broader set of exploratory analyses.

## 3 Results

### 3.1 Baseline characteristics of the randomized sample

Baseline characteristics of the three randomized groups are summarized descriptively in Table 1. These values are provided to characterize the randomized sample; no inferential baseline significance tests are used to establish equivalence between groups.

### 3.2 Executive function

Executive functions increased most in the embodied group (Table 2, Figure 2), and a significant group × time interaction was found, chi-square(6) = 78.60, *p* < 0.001 (Table 3). T0-to-T2 model-estimated changes were +10.6, +4.2, and +0.6 for the embodied, non-embodied, and control groups, respectively. In pairwise follow-up contrasts, embodied change exceeded non-embodied by 6.40 points [95% CI (3.80, 9.00), Holm *p* < 0.001] and control by 10.00 [7.40, 12.60], Holm *p* < 0.001; non-embodied exceeded control by 3.60 [1.00, 6.20], Holm *p* = 0.007. T2 embodied-versus-control d = 1.36. T1/T2/T3 within-group changes are summarized in Supplementary Table S3, and the complete pairwise contrast matrix is provided in Supplementary Table S4.

**Table 2:** Primary and secondary outcomes across four measurement periods.

Outcome	Group	T0	T1	T2	T3
Executive function	Embodied	50.2 +/− 8.0	55.0 +/− 7.6	60.8 +/− 7.3	59.5 +/− 7.4
	Non-embodied	50.0 +/− 7.9	52.5 +/− 7.7	54.2 +/− 7.6	53.8 +/− 7.7
	Control	49.8 +/− 8.1	50.1 +/− 8.0	50.4 +/− 8.0	50.3 +/− 8.1
Emotion regulation	Embodied	48.5 +/− 7.2	52.6 +/− 6.9	57.3 +/− 6.6	56.4 +/− 6.8
	Non-embodied	48.7 +/− 7.1	50.9 +/− 7.0	53.2 +/− 6.8	52.9 +/− 6.9
	Control	48.4 +/− 7.3	48.8 +/− 7.2	49.1 +/− 7.1	49.0 +/− 7.2
Synchronization	Embodied	50.1 +/− 8.5	58.0 +/− 7.9	65.1 +/− 7.4	63.2 +/− 7.6
	Non-embodied	50.0 +/− 8.4	52.3 +/− 8.2	54.1 +/− 8.0	53.6 +/− 8.1
	Control	49.9 +/− 8.6	50.1 +/− 8.5	50.2 +/− 8.4	50.1 +/− 8.5
Psychological well-being	Embodied	50.3 +/− 8.6	52.8 +/− 8.3	56.4 +/− 8.1	55.8 +/− 8.2
	Non-embodied	50.5 +/− 8.5	51.7 +/− 8.4	53.1 +/− 8.3	52.9 +/− 8.4
	Control	50.2 +/− 8.7	50.4 +/− 8.6	50.7 +/− 8.5	50.6 +/− 8.6
RMSSD, ms	Embodied	38.5 +/− 11.0	41.8 +/− 10.8	45.6 +/− 10.5	44.9 +/− 10.6
	Non-embodied	38.7 +/− 11.2	40.1 +/− 11.0	41.3 +/− 10.9	41.1 +/− 11.0
	Control	38.4 +/− 11.1	38.6 +/− 11.0	38.9 +/− 11.0	38.8 +/− 11.1
Sleep problems	Embodied	45.2 +/− 5.6	43.7 +/− 5.4	41.9 +/− 5.2	42.1 +/− 5.3
	Non-embodied	45.0 +/− 5.5	44.2 +/− 5.4	43.4 +/− 5.3	43.6 +/− 5.3
	Control	45.1 +/− 5.7	44.9 +/− 5.6	44.7 +/− 5.6	44.8 +/− 5.6

**Figure 2:**
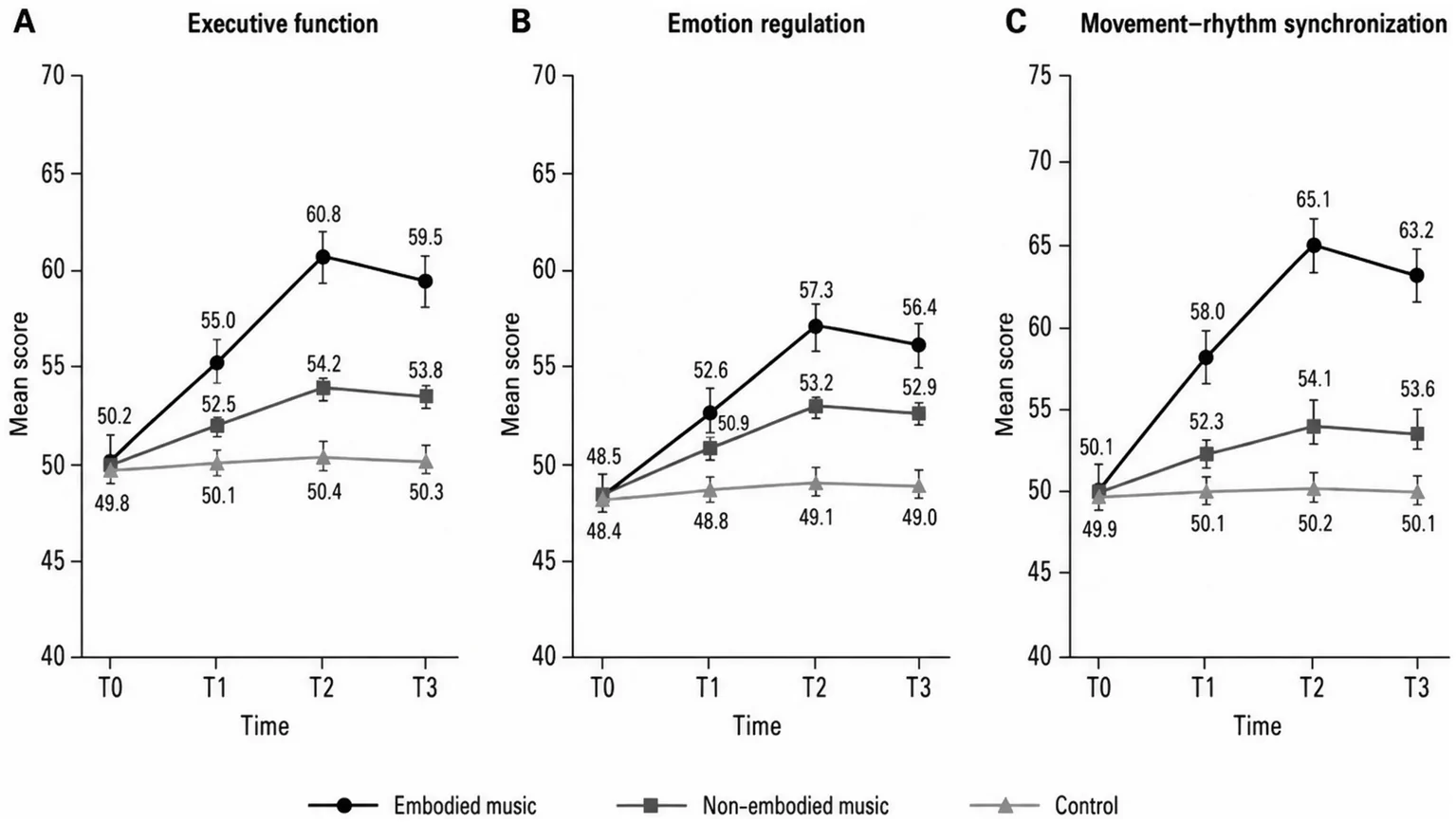
Longitudinal Changes at Four Time Points. Panels present data on executive function, emotion regulation and movement-rhythm synchronisation at baseline (T0), week 6 (T1), week 12 (T2) and 3 months post-intervention (T3). Embodied music group showed the steepest increase and retained most of the gains in follow-up.

**Table 3:** Group x time effects and principal post-intervention effect sizes.

Outcome	Group x time test	*p*	T2 embodied vs. control Cohen’s d
Movement-rhythm synchronization	Chi-square(6) = 129.30	< 0.001	1.88
Executive function	Chi-square(6) = 78.60	< 0.001	1.36
Emotion regulation	Chi-square(6) = 51.40	< 0.001	1.20
Psychological well-being	Chi-square(6) = 27.80	< 0.001	0.68
RMSSD	Chi-square(6) = 18.60	0.005	0.62
Sleep problems	Chi-square(6) = 15.20	0.019	0.52

### 3.3 Executive-function components

All three executive components showed a descriptive increase from T0 to T2 in the embodied group: inhibition (50.4 to 61.8), cognitive flexibility (50.3 to 61.1) and working memory (49.9 to 58.7) (Table 4), and smaller changes were observed in non-embodied music and controls. As the goal of inference was an interactive group-time effect, only the individual component results were reported.

**Table 4:** Executive-function component scores at baseline and post-intervention.

Outcome	Group	T0	T2	Change
Inhibitory control	Embodied	50.4	61.8	+11.4
	Non-embodied	50.2	54.7	+4.5
	Control	50.0	50.6	+0.6
Working memory	Embodied	49.9	58.7	+8.8
	Non-embodied	50.1	53.5	+3.4
	Control	49.8	50.3	+0.5
Cognitive flexibility	Embodied	50.3	61.1	+10.8
	Non-embodied	49.7	54.3	+4.6
	Control	49.9	50.2	+0.3

### 3.4 Emotion regulation

Emotion regulation showed a significant group × time interaction, chi-square(6) = 51.40, *p* < 0.001 (Tables 2, 3; Figure 2). From T0 to T2, embodied change exceeded non-embodied by 4.30 points (95% CI [2.00, 6.60], Holm *p* < 0.001) and control by 8.10 [5.70, 10.50], Holm *p* < 0.001; non-embodied also exceeded control by 3.80 [1.45, 6.15], Holm *p* = 0.002. T2 embodied-versus-control *d* = 1.20. Within embodied music, T1/T2/T3 changed by +4.10/+8.80/+7.90 (Supplementary Table S3).

### 3.5 Movement-rhythm synchronization

Movement-rhythm synchronisation produced the largest effect (Group × Time chi-square(6) = 129.30, *p* < 0.001; Tables 2, 3; Figure 2). T0-to-T2 changes were +15.0, +4.1, and +0.3 in the embodied, non-embodied, and control groups, respectively. Embodied change exceeded non-embodied by 10.90 points (95% CI [8.20, 13.60], Holm *p* < 0.001) and control by 14.70 [12.00, 17.40], Holm *p* < 0.001; the non-embodied-versus-control contrast was 3.80 [1.10, 6.50], Holm *p* = 0.006. T2 embodied-versus-control d = 1.88. Within-group contrasts are given in Supplementary Table S3 and participant-level trajectories in Supplementary Figure S1.

### 3.6 Psychological well-being

Psychological well-being showed a significant Group × Time interaction, chi-square(6) = 27.80, *p* < 0.001 (Tables 2,3). From T0 to T2, embodied change exceeded non-embodied by 3.50 points (95% CI [1.10, 5.90], Holm *p* = 0.009) and control by 5.60 [3.20, 8.00], Holm *p* < 0.001. The non-embodied-versus-control contrast was 2.10 [−0.30, 4.50], Holm *p* = 0.086. The descriptive T2 embodied-versus-control effect size was d = 0.68. Within-group contrasts are reported in Supplementary Table S3.

### 3.7 Heart rate variability

RMSSD showed a significant Group × Time interaction, chi-square(6) = 18.60, *p* = 0.005 (Tables 2,3). From T0 to T2, embodied change exceeded non-embodied by 4.50 ms [95% CI (1.10, 7.90), Holm *p* = 0.019] and control by 6.60 ms [3.20, 10.00], Holm *p* < 0.001. The non-embodied-versus-control contrast was 2.10 ms [−1.30, 5.50], Holm *p* = 0.226. The descriptive T2 embodied-versus-control effect size was d = 0.62. Within-group contrasts are reported in Supplementary Table S3.

### 3.8 Sleep problems

Sleep effects were smaller [Group × Time chi-square(6) = 15.20, *p* = 0.019; Tables 2,3]. From T0 to T2, embodied change did not significantly exceed non-embodied change after the three-comparison Holm adjustment [−1.70 points, 95% CI (−3.60, 0.20), *p* = 0.159] but did exceed control [−2.90 (−4.80, −1.00), *p* = 0.008]. The non-embodied-versus-control contrast was −1.20 [−3.10, 0.70], Holm *p* = 0.216. The absolute descriptive T2 embodied-versus-control effect size was d = 0.52. Within-group contrasts are reported in Supplementary Table S3.

### 3.9 Complete longitudinal outcome pattern

A high score indicates good performance of the executive function, emotion regulation, synchrony, and psychological well-being. Higher sleep-problem scores indicate more sleep problems. Available outcome data by group (embodied/non-embodied/control) were T0: 60/60/60; T1: 59/59/58; T2: 58/57/57; and T3: 56/55/55. Mixed-effects models were employed for all 180 randomised subjects with repeated observations.

Table 2 and Figure 2 show the largest T0-to-T2 separation for synchronization, executive function, and emotion regulation, with modest attenuation but persistent differences at T3. Changes in the non-embodied condition were smaller, whereas the control group remained comparatively stable. Inferential conclusions are based on the mixed-effects models and model-derived contrasts reported above.

### 3.10 Summary of longitudinal mixed-effects models

Table 3 summarizes the omnibus Group × Time tests and the descriptive T2 embodied-versus-control standardized effect sizes. Synchronization, executive function, and emotion regulation showed the largest standardized differences, but these values should not be interpreted as evidence of an ordered causal mechanism. Complete three-way pairwise follow-up contrasts are presented in Supplementary Table S4.

Supplementary Figure S1 displays participant-level longitudinal variability, and Supplementary Table S2 reports sample-specific reliability estimates.

### 3.11 Maintenance of intervention effects

At T3, embodied-group executive function, emotion regulation and synchronisation remained at +9.3, +7.9 and +13.1 points above baseline respectively, showing considerable short-term retention after the cessation of training (Table 2).

### 3.12 Associations among change variables

Using T0-to-T2 change scores, synchronization change correlated with executive-function change (*r* = 0.48), emotion-regulation change (*r* = 0.36), and well-being change (*r* = 0.27). Executive-function change correlated with emotion-regulation change (*r* = 0.44) and well-being change (*r* = 0.33), and emotion-regulation change correlated with well-being change (*r* = 0.41); all *p* < 0.01. These moderate associations are exploratory and do not establish temporal direction or mediation.

### 3.13 Exploratory indirect-effect analyses

In the first exploratory indirect-effect model, embodied versus non-embodied music predicted T0-to-T2 synchronization improvement (standardized beta = 0.57, SE = 0.07, *p* < 0.001), which was associated with executive-function improvement (beta = 0.39, SE = 0.07, *p* < 0.001). The standardized indirect effect was 0.22, bootstrap 95% CI [0.14, 0.32] (5,000 resamples). In the broader exploratory model, standardized paths were embodied music > synchronization beta = 0.57, synchronization > executive function beta = 0.39, executive function > emotion regulation beta = 0.34, and emotion regulation > T3 well-being beta = 0.29 (all *p* < 0.001). The full serial indirect effect was 0.022, bootstrap 95% CI [0.009, 0.043]; the synchronization/executive-function indirect association with emotion regulation was 0.076 [0.038, 0.128]. The total association with T3 well-being was beta = 0.34, *p* < 0.001, and the residual direct association was beta = 0.16, *p* = 0.041. Because the proposed mediators overlapped temporally and were not experimentally manipulated, these estimates are exploratory statistical associations and do not establish a serial causal sequence (Figure 3).

**Figure 3:**
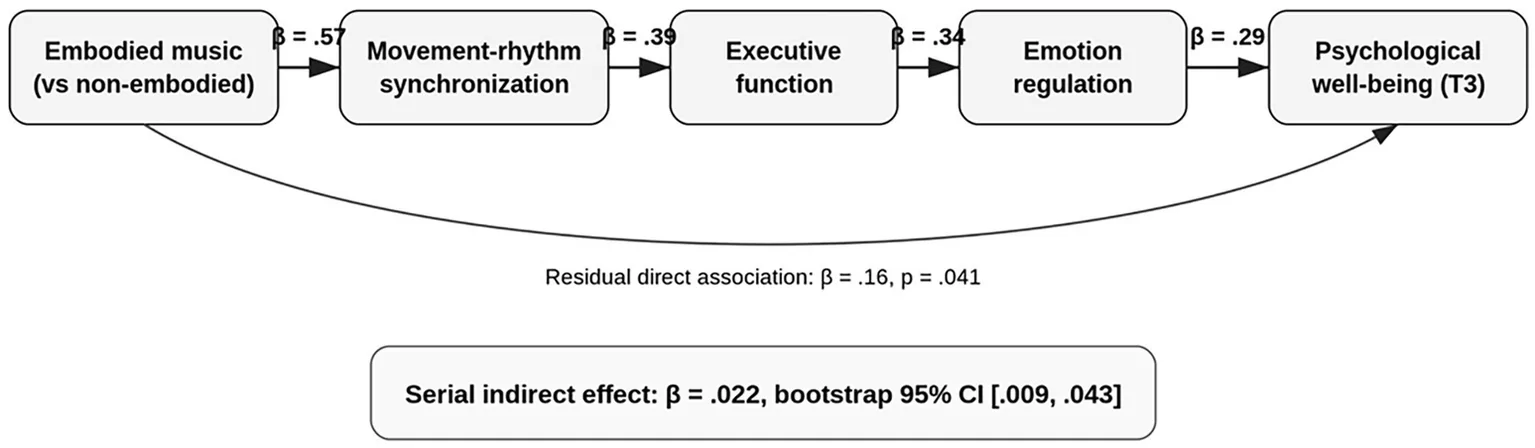
Exploratory indirect-effect model linking embodied music with later psychological well-being. Standardized path coefficients are shown above arrows. The reported serial indirect effect through movement-rhythm synchronization, executive function, and emotion regulation was beta = 0.022, bootstrap 95% CI [0.009, 0.043]. Because the proposed mediators overlap in time, the figure is descriptive of the fitted statistical model and does not establish temporal or causal mediation.

## 4 Discussion

This randomized trial examined whether structured bodily engagement added to the effects of music participation. Embodied music showed larger longitudinal gains in executive function, synchronization, and emotion regulation than the comparison conditions, while psychological well-being, RMSSD, and sleep showed smaller changes. The overall pattern is compatible with stronger effects on more proximal outcomes, but it does not by itself establish an ordered developmental mechanism Figure 4.

**Figure 4:**
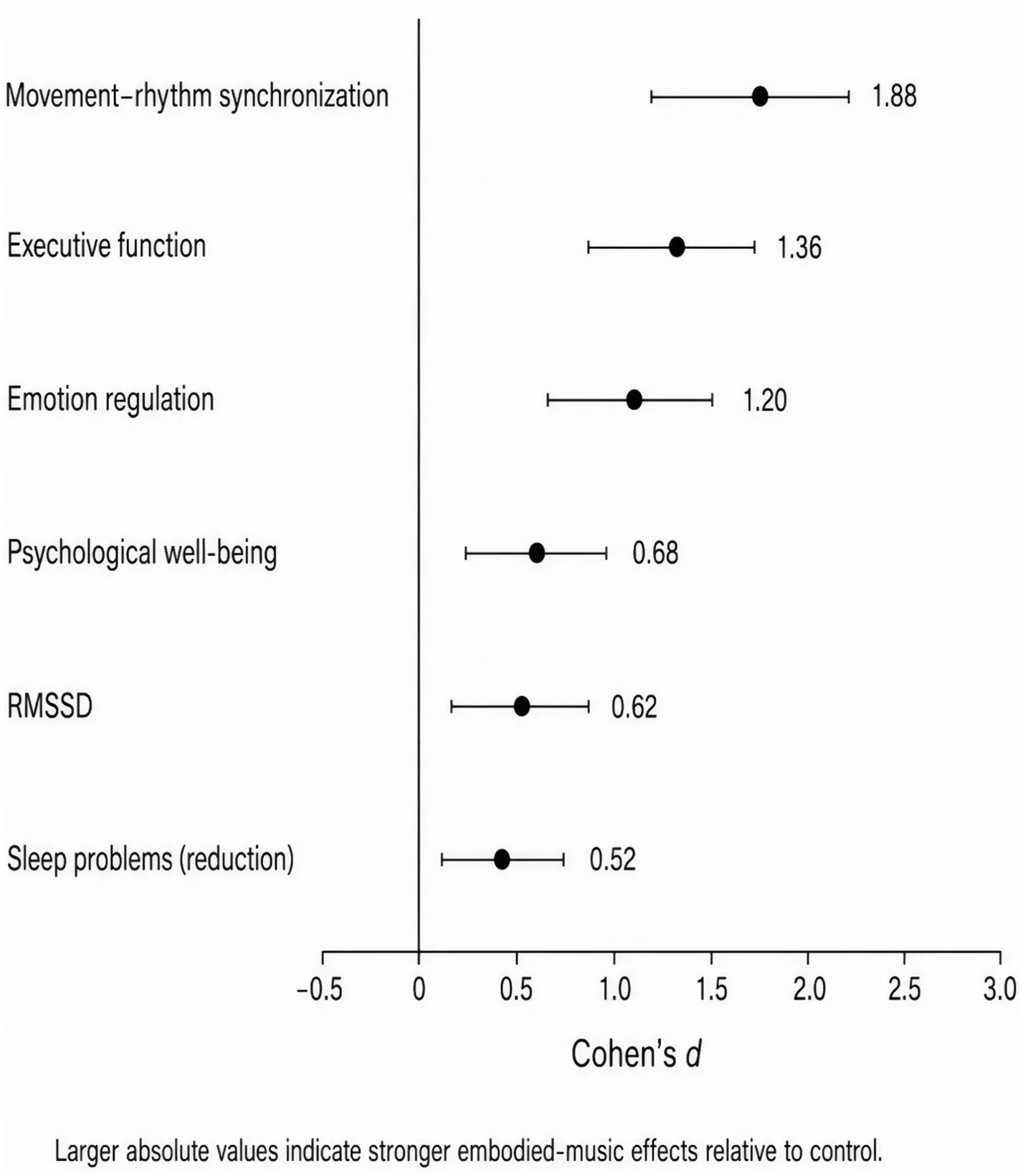
Standardised effects at post-intervention for embodied music vs. usual-activity control. Point Estimates are Cohen’s d. The largest effects were observed in the areas of movement-rhythm synchronisation, executive function and emotion regulation; psychological well-being, RMSSD and sleep showed smaller effects.

### 4.1 Embodied music and executive functions

The executive-function findings extend evidence linking music participation with cognitive control (Moreno et al., 2011; Jaschke et al., 2018; Frischen et al., 2019). Because the two active conditions were matched on musical exposure, instructor contact, social participation, and intervention dose, the additional 6.4-point T0-to-T2 gain in the embodied group is consistent with an incremental effect of the structured bodily component rather than music exposure alone.

The embodied intervention repeatedly required children to stop or reverse actions, suppress prepotent responses, and switch rules in response to changing musical cues, providing a plausible basis for the observed inhibition and flexibility differences. Working-memory changes were smaller, which is compatible with the fact that working memory was engaged indirectly through retention of rhythmic and movement rules rather than being trained as explicitly. This interpretation is consistent with the view that executive control can benefit from activities that repeatedly challenge cognition while engaging physical and social participation (Diamond and Lee, 2011).

### 4.2 Movement-rhythm synchronization as a proximal outcome

Synchronization showed the largest between-group standardized difference, which is consistent with its close proximity to the activities practiced during the intervention. Accurate synchronization involves auditory timing, prediction, motor planning, and error correction (Repp and Su, 2013), consistent with embodied accounts of perception-action coupling (Maes et al., 2014; Phillips-Silver and Trainor, 2005). However, the T2 effect (*d* = 1.88) is unusually large for a school-based behavioral intervention and should be interpreted cautiously and independently replicated.

Synchronization change was statistically associated with part of the embodied-versus-non-embodied difference in executive-function change in the exploratory indirect-effect analysis. Because the variables were not experimentally manipulated or fully separated in time, this result should be interpreted as being consistent with, rather than demonstrating, a perception-action pathway.

### 4.3 From executive control to emotion regulation

Executive function and emotion regulation are distinct but related domains: regulating affect often requires inhibition, attentional shifting, and maintenance of regulatory goals. The observed T0-to-T2 change correlation (*r* = 0.44) is therefore compatible with shared self-regulatory processes but does not establish directionality or mediation. Group synchronization may also involve social monitoring, coordination, delayed responding, and recovery from joint errors, although these possibilities were not independently tested in the present study.

This interpretation is compatible with evidence linking joint music making with cooperation and empathy (Kirschner and Tomasello, 2010; Rabinowitch et al., 2013). The non-embodied music group also showed greater T0-to-T2 improvement in emotion regulation than the usual-activity control (difference in change = 3.80, 95% CI [1.30, 6.30], Holm *p* = 0.006), while embodied music showed an additional 4.30-point advantage over the non-embodied condition. These comparisons suggest that structured music participation may relate to improved regulation, with an additional benefit associated with the embodied condition.

### 4.4 Psychological well-being and psychophysiological regulation

Psychological well-being changed less than synchronization, executive function, and emotion regulation, which is plausible because it is influenced by many experiences beyond a 12-week program. RMSSD showed a similarly more modest pattern: from T0 to T2, the embodied group increased by 7.1 ms, compared with 2.6 ms in the non-embodied group and 0.5 ms in controls. Given the sensitivity of HRV to recording conditions (Laborde et al., 2017), these findings are best interpreted as convergent physiological observations rather than evidence of change in a single regulatory mechanism. Sleep, which was not directly trained, showed the smallest standardized difference.

### 4.5 Pattern of effects across outcomes

The observed ordering of standardized effects is descriptively compatible with stronger effects on outcomes that were more proximal to the intervention, but it does not establish a layered developmental mechanism. T2 embodied-versus-control effects were *d* = 1.88 for synchronization, 1.36 for executive function, 1.20 for emotion regulation, 0.68 for well-being, 0.62 for RMSSD, and 0.52 for sleep. These effect sizes are influenced by measurement reliability, scale dispersion, task similarity, and outcome sensitivity. Interpretation therefore relies on the longitudinal model contrasts, raw means/SDs, reliability estimates (Supplementary Table S2), participant trajectories (Supplementary Figure S1), and sensitivity analyses (Supplementary Tables S3, S4), rather than on rank ordering alone.

The exploratory serial indirect-effect model was statistically consistent with the hypothesized sequence, but the indirect effect was small (beta = 0.022) and the residual direct association with well-being remained significant. Because the proposed mediators were temporally overlapping and not experimentally manipulated, we do not interpret this pattern as evidence that partial mediation or a causal serial mechanism occurred. Enjoyment, physical activity, peer interaction, self-efficacy, competence, belonging, and other unmeasured processes may also contribute to the observed associations.

### 4.6 Maintenance after intervention cessation

At 3-month follow-up, executive function, emotion regulation, and synchronization remained above baseline but were modestly lower than at T2. This pattern is consistent with partial maintenance of the observed gains and motivates future studies comparing continued training, booster sessions, and withdrawal of structured practice.

### 4.7 Theoretical implications

The findings have three theoretical implications. First, they are consistent with the possibility that bodily engagement contributes additional information beyond auditory participation alone. Second, they support considering executive and emotional regulation within a related self-regulatory framework while avoiding causal claims about their ordering. Third, they emphasize the importance of distinguishing directly trained outcomes from more distal outcomes rather than assuming uniform intervention effects across domains.

### 4.8 Practical applications

The program used activities such as clapping, stepping, body percussion, movement inhibition, rule reversal, and peer synchronization that can be incorporated into school music, movement, or extracurricular sessions. The randomized comparisons suggest that the mode of participation, including structured perception-action coupling, may be relevant to intervention effects; however, implementation claims should be interpreted in light of the study’s measurement and replication limitations.

### 4.9 Limitations

Several limitations qualify interpretation. The sample comprised typically developing 8-11-year-old children from six schools, limiting generalizability to other ages, clinical populations, and educational contexts. Complete double blinding was not feasible: outcome assessors were blinded, but intervention instructors and fidelity observers could identify condition from session content. The embodied program combined synchronization, physical movement, inhibition, switching, and social coordination, so component-specific causal effects cannot be isolated. Questionnaire-based well-being and sleep outcomes remain vulnerable to reporting bias. RMSSD is sensitive to respiration, posture, recent activity, recording time, device characteristics, and preprocessing decisions; the current manuscript does not report all of these technical acquisition details.

The exploratory indirect-effect analyses did not separate all proposed mediators across independent time points and therefore cannot establish temporal or causal mediation. Several standardized effects, particularly for synchronization and executive function, are large relative to many school-based interventions and therefore warrant cautious interpretation and independent replication. The limited number of school-by-condition cohorts also constrains higher-level random-effects inference. In addition, the missing-at-random assumption cannot be verified from the observed data alone, and the current report does not include sensitivity analyses under alternative missing-not-at-random mechanisms. These limitations should be considered when interpreting the magnitude and generalizability of the findings.

## 5 Conclusion

In this three-arm randomized trial, embodied music was associated with larger longitudinal gains in movement-rhythm synchronization, executive function, and emotion regulation than the comparison conditions, with smaller changes in psychological well-being, RMSSD, and sleep. Compared with non-embodied music, the embodied condition showed greater T0-to-T2 change in the primary outcomes and synchronization. Exploratory indirect-effect analyses were statistically consistent with associations among synchronization, executive control, emotion regulation, and later well-being, but temporal overlap among the proposed mediators precludes serial causal inference. The large proximal effects warrant independent replication before broad generalization.

## Data Availability

The raw data supporting the conclusions of this article will be made available by the authors, without undue reservation.
