# Supplementary material for: Effects of an embodied music intervention on executive function, emotion regulation and psychophysiological well-being in children: the role of movement-rhythm synchronization and self-regulation
Source: Front Psychol. 2026 Oct 20;17:1988460. doi: 10.3389/fpsyg.2026.1988460 (PMC13727424; doi:10.3389/fpsyg.2026.1988460)
Supplement: Supplementary file 1 [file Supplementary_file_1.docx]

**Supplementary Materials**

*Effects of an Embodied Music Intervention on Executive Function, Emotion Regulation and Psychophysiological Well-being in Children: The Role of Movement-Rhythm Synchronization and Self-Regulation*

**Supplementary Table S1. School-specific randomized allocation**

| **School** | **Randomized total** | **Embodied music** | **Non-embodied music** | **Usual activity** |
| --- | --- | --- | --- | --- |
| School 1 | 32 | 11 | 10 | 11 |
| School 2 | 28 | 9 | 10 | 9 |
| School 3 | 31 | 10 | 11 | 10 |
| School 4 | 29 | 10 | 9 | 10 |
| School 5 | 30 | 10 | 10 | 10 |
| School 6 | 30 | 10 | 10 | 10 |

*Note.* Randomization was stratified within school and sex. Totals across the six schools were 60 children per study arm (N = 180).

**Supplementary Table S2. Sample-specific measurement reliability**

| **Measure** | **Coefficient** | **T0** | **T1** | **T2** | **T3** |
| --- | --- | --- | --- | --- | --- |
| Emotion Regulation Checklist | alpha / omega | .88 / .89 | .89 / .90 | .90 / .91 | .89 / .90 |
| KIDSCREEN-10 | alpha / omega | .84 / .85 | .85 / .86 | .86 / .87 | .86 / .87 |
| Children's Sleep Habits Questionnaire | alpha / omega | .81 / .82 | .82 / .83 | .83 / .84 | .82 / .83 |
| Executive-function composite | McDonald's omega | .82 | .84 | .86 | .85 |
| Flanker IES | split-half | .83 | .84 | .85 | .84 |
| Backward digit span | KR-20 | .79 | .80 | .82 | .81 |
| Rule-switching IES | split-half | .81 | .82 | .84 | .83 |
| Synchronization composite | split-half | .86 | .87 | .89 | .88 |

*Note.* Alpha = Cronbach's alpha; omega = McDonald's omega; KR-20 = Kuder-Richardson coefficient. Split-half coefficients were Spearman-Brown corrected. IES = inverse-efficiency score.

**Supplementary Table S3. Model-derived within-group time contrasts**

| **Outcome** | **Group** | **Contrast** | **Estimate** | **95% CI** | **Adjusted p** |
| --- | --- | --- | --- | --- | --- |
| Executive function | Embodied | T1-T0 | 4.80 | [3.03, 6.57] | < .001 |
| Executive function | Embodied | T2-T0 | 10.60 | [8.84, 12.36] | < .001 |
| Executive function | Embodied | T3-T0 | 9.30 | [7.50, 11.10] | < .001 |
| Executive function | Non-embodied | T1-T0 | 2.50 | [0.74, 4.26] | .006 |
| Executive function | Non-embodied | T2-T0 | 4.20 | [2.41, 5.99] | < .001 |
| Executive function | Non-embodied | T3-T0 | 3.80 | [1.97, 5.63] | < .001 |
| Executive function | Control | T1-T0 | 0.30 | [-1.54, 2.14] | 1.000 |
| Executive function | Control | T2-T0 | 0.60 | [-1.25, 2.45] | 1.000 |
| Executive function | Control | T3-T0 | 0.50 | [-1.40, 2.40] | 1.000 |
| Emotion regulation | Embodied | T1-T0 | 4.10 | [2.38, 5.82] | < .001 |
| Emotion regulation | Embodied | T2-T0 | 8.80 | [7.09, 10.51] | < .001 |
| Emotion regulation | Embodied | T3-T0 | 7.90 | [6.14, 9.66] | < .001 |
| Emotion regulation | Non-embodied | T1-T0 | 2.20 | [0.48, 3.92] | .013 |
| Emotion regulation | Non-embodied | T2-T0 | 4.50 | [2.77, 6.23] | < .001 |
| Emotion regulation | Non-embodied | T3-T0 | 4.20 | [2.43, 5.97] | < .001 |
| Emotion regulation | Control | T1-T0 | 0.40 | [-1.39, 2.19] | 1.000 |
| Emotion regulation | Control | T2-T0 | 0.70 | [-1.09, 2.49] | 1.000 |
| Emotion regulation | Control | T3-T0 | 0.60 | [-1.24, 2.44] | 1.000 |
| Synchronization | Embodied | T1-T0 | 7.90 | [5.75, 10.05] | < .001 |
| Synchronization | Embodied | T2-T0 | 15.00 | [12.89, 17.11] | < .001 |
| Synchronization | Embodied | T3-T0 | 13.10 | [10.93, 15.27] | < .001 |
| Synchronization | Non-embodied | T1-T0 | 2.30 | [0.13, 4.47] | .038 |
| Synchronization | Non-embodied | T2-T0 | 4.10 | [1.92, 6.28] | .001 |
| Synchronization | Non-embodied | T3-T0 | 3.60 | [1.36, 5.84] | .004 |
| Synchronization | Control | T1-T0 | 0.20 | [-2.05, 2.45] | 1.000 |
| Synchronization | Control | T2-T0 | 0.30 | [-1.96, 2.56] | 1.000 |
| Synchronization | Control | T3-T0 | 0.20 | [-2.12, 2.52] | 1.000 |
| Well-being | Embodied | T1-T0 | 2.50 | [0.43, 4.57] | .019 |
| Well-being | Embodied | T2-T0 | 6.10 | [4.04, 8.16] | < .001 |
| Well-being | Embodied | T3-T0 | 5.50 | [3.39, 7.61] | < .001 |
| Well-being | Non-embodied | T1-T0 | 1.20 | [-0.86, 3.26] | .249 |
| Well-being | Non-embodied | T2-T0 | 2.60 | [0.51, 4.69] | .047 |
| Well-being | Non-embodied | T3-T0 | 2.40 | [0.26, 4.54] | .057 |
| Well-being | Control | T1-T0 | 0.20 | [-1.93, 2.33] | 1.000 |
| Well-being | Control | T2-T0 | 0.50 | [-1.64, 2.64] | 1.000 |
| Well-being | Control | T3-T0 | 0.40 | [-1.79, 2.59] | 1.000 |
| RMSSD | Embodied | T1-T0 | 3.30 | [0.84, 5.76] | .010 |
| RMSSD | Embodied | T2-T0 | 7.10 | [4.64, 9.56] | < .001 |
| RMSSD | Embodied | T3-T0 | 6.40 | [3.89, 8.91] | < .001 |
| RMSSD | Non-embodied | T1-T0 | 1.40 | [-1.11, 3.91] | .269 |
| RMSSD | Non-embodied | T2-T0 | 2.60 | [0.06, 5.14] | .136 |
| RMSSD | Non-embodied | T3-T0 | 2.40 | [-0.20, 5.00] | .140 |
| RMSSD | Control | T1-T0 | 0.20 | [-2.32, 2.72] | 1.000 |
| RMSSD | Control | T2-T0 | 0.50 | [-2.04, 3.04] | 1.000 |
| RMSSD | Control | T3-T0 | 0.40 | [-2.20, 3.00] | 1.000 |
| Sleep problems | Embodied | T1-T0 | -1.50 | [-2.84, -0.16] | .029 |
| Sleep problems | Embodied | T2-T0 | -3.30 | [-4.63, -1.97] | < .001 |
| Sleep problems | Embodied | T3-T0 | -3.10 | [-4.47, -1.73] | < .001 |
| Sleep problems | Non-embodied | T1-T0 | -0.80 | [-2.13, 0.53] | .234 |
| Sleep problems | Non-embodied | T2-T0 | -1.60 | [-2.94, -0.26] | .061 |
| Sleep problems | Non-embodied | T3-T0 | -1.40 | [-2.77, -0.03] | .090 |
| Sleep problems | Control | T1-T0 | -0.20 | [-1.59, 1.19] | 1.000 |
| Sleep problems | Control | T2-T0 | -0.40 | [-1.81, 1.01] | 1.000 |
| Sleep problems | Control | T3-T0 | -0.30 | [-1.73, 1.13] | 1.000 |

*Note.* Estimates are model-derived changes from baseline within each group. Confidence intervals are pointwise model-based 95% CIs and were not multiplicity-adjusted; reported p values were Holm adjusted across the three postbaseline comparisons within each outcome-by-group family. Positive estimates indicate improvement except for sleep problems, where negative estimates indicate fewer problems.

**Supplementary Table S4. Pairwise Group x Time contrasts across follow-up occasions**

| **Outcome** | **Time contrast** | **Comparison** | **Difference in change** | **95% CI** | **Holm-adjusted p** |
| --- | --- | --- | --- | --- | --- |
| Executive function | T1-T0 | Embodied vs Non-embodied | 2.30 | [-0.17, 4.77] | .143 |
| Executive function | T1-T0 | Embodied vs Control | 4.50 | [1.97, 7.03] | .001 |
| Executive function | T1-T0 | Non-embodied vs Control | 2.20 | [-0.30, 4.70] | .143 |
| Executive function | T2-T0 | Embodied vs Non-embodied | 6.40 | [3.80, 9.00] | < .001 |
| Executive function | T2-T0 | Embodied vs Control | 10.00 | [7.40, 12.60] | < .001 |
| Executive function | T2-T0 | Non-embodied vs Control | 3.60 | [1.00, 6.20] | .007 |
| Executive function | T3-T0 | Embodied vs Non-embodied | 5.50 | [2.96, 8.04] | < .001 |
| Executive function | T3-T0 | Embodied vs Control | 8.80 | [6.21, 11.39] | < .001 |
| Executive function | T3-T0 | Non-embodied vs Control | 3.30 | [0.73, 5.87] | .012 |
| Emotion regulation | T1-T0 | Embodied vs Non-embodied | 1.90 | [-0.51, 4.31] | .252 |
| Emotion regulation | T1-T0 | Embodied vs Control | 3.70 | [1.24, 6.16] | .009 |
| Emotion regulation | T1-T0 | Non-embodied vs Control | 1.80 | [-0.64, 4.24] | .252 |
| Emotion regulation | T2-T0 | Embodied vs Non-embodied | 4.30 | [2.00, 6.60] | < .001 |
| Emotion regulation | T2-T0 | Embodied vs Control | 8.10 | [5.70, 10.50] | < .001 |
| Emotion regulation | T2-T0 | Non-embodied vs Control | 3.80 | [1.45, 6.15] | .002 |
| Emotion regulation | T3-T0 | Embodied vs Non-embodied | 3.70 | [1.23, 6.17] | .007 |
| Emotion regulation | T3-T0 | Embodied vs Control | 7.30 | [4.78, 9.82] | < .001 |
| Emotion regulation | T3-T0 | Non-embodied vs Control | 3.60 | [1.10, 6.10] | .007 |
| Synchronization | T1-T0 | Embodied vs Non-embodied | 5.60 | [2.58, 8.62] | < .001 |
| Synchronization | T1-T0 | Embodied vs Control | 7.70 | [4.62, 10.78] | < .001 |
| Synchronization | T1-T0 | Non-embodied vs Control | 2.10 | [-0.95, 5.15] | .177 |
| Synchronization | T2-T0 | Embodied vs Non-embodied | 10.90 | [8.20, 13.60] | < .001 |
| Synchronization | T2-T0 | Embodied vs Control | 14.70 | [12.00, 17.40] | < .001 |
| Synchronization | T2-T0 | Non-embodied vs Control | 3.80 | [1.10, 6.50] | .006 |
| Synchronization | T3-T0 | Embodied vs Non-embodied | 9.50 | [6.41, 12.59] | < .001 |
| Synchronization | T3-T0 | Embodied vs Control | 12.90 | [9.76, 16.04] | < .001 |
| Synchronization | T3-T0 | Non-embodied vs Control | 3.40 | [0.29, 6.51] | .032 |
| Well-being | T1-T0 | Embodied vs Non-embodied | 1.30 | [-1.59, 4.19] | .764 |
| Well-being | T1-T0 | Embodied vs Control | 2.30 | [-0.64, 5.24] | .366 |
| Well-being | T1-T0 | Non-embodied vs Control | 1.00 | [-1.92, 3.92] | .764 |
| Well-being | T2-T0 | Embodied vs Non-embodied | 3.50 | [1.10, 5.90] | .009 |
| Well-being | T2-T0 | Embodied vs Control | 5.60 | [3.20, 8.00] | < .001 |
| Well-being | T2-T0 | Non-embodied vs Control | 2.10 | [-0.30, 4.50] | .086 |
| Well-being | T3-T0 | Embodied vs Non-embodied | 3.10 | [0.12, 6.08] | .085 |
| Well-being | T3-T0 | Embodied vs Control | 5.10 | [2.09, 8.11] | .003 |
| Well-being | T3-T0 | Non-embodied vs Control | 2.00 | [-0.99, 4.99] | .191 |
| RMSSD | T1-T0 | Embodied vs Non-embodied | 1.90 | [-1.59, 5.39] | .572 |
| RMSSD | T1-T0 | Embodied vs Control | 3.10 | [-0.39, 6.59] | .245 |
| RMSSD | T1-T0 | Non-embodied vs Control | 1.20 | [-2.29, 4.69] | .572 |
| RMSSD | T2-T0 | Embodied vs Non-embodied | 4.50 | [1.10, 7.90] | .019 |
| RMSSD | T2-T0 | Embodied vs Control | 6.60 | [3.20, 10.00] | < .001 |
| RMSSD | T2-T0 | Non-embodied vs Control | 2.10 | [-1.30, 5.50] | .226 |
| RMSSD | T3-T0 | Embodied vs Non-embodied | 4.00 | [0.42, 7.58] | .057 |
| RMSSD | T3-T0 | Embodied vs Control | 6.00 | [2.42, 9.58] | .003 |
| RMSSD | T3-T0 | Non-embodied vs Control | 2.00 | [-1.58, 5.58] | .274 |
| Sleep problems | T1-T0 | Embodied vs Non-embodied | -0.70 | [-2.57, 1.17] | .938 |
| Sleep problems | T1-T0 | Embodied vs Control | -1.30 | [-3.22, 0.62] | .536 |
| Sleep problems | T1-T0 | Non-embodied vs Control | -0.60 | [-2.50, 1.29] | .938 |
| Sleep problems | T2-T0 | Embodied vs Non-embodied | -1.70 | [-3.60, 0.20] | .159 |
| Sleep problems | T2-T0 | Embodied vs Control | -2.90 | [-4.80, -1.00] | .008 |
| Sleep problems | T2-T0 | Non-embodied vs Control | -1.20 | [-3.10, 0.70] | .216 |
| Sleep problems | T3-T0 | Embodied vs Non-embodied | -1.70 | [-3.62, 0.22] | .172 |
| Sleep problems | T3-T0 | Embodied vs Control | -2.80 | [-4.76, -0.84] | .014 |
| Sleep problems | T3-T0 | Non-embodied vs Control | -1.10 | [-3.04, 0.84] | .266 |

Note. Values are reported as point estimates with 95% confidence intervals. Two-sided p values were Holm-adjusted across all three pairwise group comparisons within each outcome × follow-up family. Negative estimates for sleep problems favor the first named group because higher scores indicate greater sleep difficulty.

**Supplementary Figure S1. Participant-level longitudinal trajectories**

Figure S1. Participant-level longitudinal trajectories for executive function, emotion regulation, and movement-rhythm synchronization. Thin lines represent individual children with available observations; the thick line represents the group mean and the shaded band the 95% confidence interval. The distributions show appreciable between-child variability within each condition across the four assessment occasions.
